# β-Adrenergic Agonist and Antagonist Regulation of Autophagy in HepG2 Cells, Primary Mouse Hepatocytes, and Mouse Liver

**DOI:** 10.1371/journal.pone.0098155

**Published:** 2014-06-20

**Authors:** Benjamin L. Farah, Rohit A. Sinha, Yajun Wu, Brijesh K. Singh, Jin Zhou, Boon-Huat Bay, Paul M. Yen

**Affiliations:** 1 Cardiovascular and Metabolic Disorders Program, Duke-NUS Graduate Medical School Singapore, Singapore; 2 Department of Anatomy, Yong Loo Lin School of Medicine, National University of Singapore, Singapore; 3 Sarah W. Stedman Nutrition and Metabolism Center, Departments of Medicine and Pharmacology and Cancer Biology, Duke University Medical Center, Durham, North Carolina, United States of America; Institute of Hepatology, Foundation for Liver Research, United Kingdom

## Abstract

Autophagy recently has been shown to be involved in normal hepatic function and in pathological conditions such as non-alcoholic fatty liver disease. Adrenergic signalling also is an important regulator of hepatic metabolism and function. However, currently little is known about the potential role of adrenergic signaling on hepatic autophagy, and whether the β-adrenergic receptor itself may be a key regulator of autophagy. To address these issues, we investigated the actions of the β_2_-adrenergic receptor agonist, clenbuterol on hepatic autophagy. Surprisingly, we found that clenbuterol stimulated autophagy and autophagic flux in hepatoma cells, primary hepatocytes and *in vivo*. Similar effects also were observed with epinephrine treatment. Interestingly, propranolol caused a late block in autophagy in the absence and presence of clenbuterol, both in cell culture and *in vivo*. Thus, our results demonstrate that the β_2_- adrenergic receptor is a key regulator of hepatic autophagy, and that the β-blocker propranolol can independently induce a late block in autophagy.

## Introduction

Macroautophagy (hereafter referred to as autophagy), is a bulk degradative process in which cytoplasmic elements are engulfed in membrane-bound organelles, autophagosomes, that are subsequently digested in autolysosomes generated by the fusion of autophagosomes and lysosomes [1]. Derangements in autophagy have been linked to many human disorders, including cancer, neurodegenerative disorders, and metabolic diseases [2]. Autophagy, in the whole liver, was shown to degrade proteins to generate amino acids used for energy during fasting [3] and to be essential for basal and thyroid hormone-stimulated fatty acid β-oxidation [4], [5]. Loss of autophagy also has been linked to hepatic injury and tumour development via Nuclear Factor (erythroid-derived-2)-like-2 (Nrf2) pathway activation [6]. In contrast, autophagy was linked to increased hepatic stellate cell activation and hepatic fibrosis [7]–[9]. Thus, autophagy may have beneficial or deleterious effects on the liver depending on the hepatic cell type involved, and the disease context.

β-adrenergic receptor agonists are used clinically to treat airway disorders such as asthma [10]. Additionally, β-adrenergic receptor antagonists, known as “β-blockers” often are used to treat hypertension and ischemic heart disease [11]. Elucidation of the β-adrenergic receptor signalling mechanisms has deepened our understanding of many fundamental physiological and cellular processes. However, despite extensive clinical experience with drugs that act via the β-adrenergic receptor and the characterization of the molecular properties of the receptor and its downstream signalling pathway, little is known about the effects of the β-adrenergic receptor on hepatic autophagy. Initial reports suggested that adrenergic stimulation and cAMP mediated signalling pathway activation inhibited autophagy [12]–[14]; however, later studies showed that adrenergic stimulation induced autophagy [15]–[18]. In particular, adrenergic agonists caused glycogen-specific autophagy in newborn rat liver [15] and β_2_-adrenergic agonists stimulated autophagy in cardiac fibroblasts that contributes to increased degradation of abnormal collagen deposits [16]. Additionally, mice that lacked epinephrine displayed decreased hepatic autophagy [18]. Given these conflicting findings in different tissues and model systems, and the central role of epinephrine in hepatic metabolism, we investigated the effect of β-adrenergic agonists and antagonists on hepatocyte autophagy. Using multiple techniques, we found that the long-acting β_2_-adrenergic agonist, clenbuterol (Clen) [19], increased autophagic flux in human hepatoma cells, mouse primary hepatocytes, and mouse livers. In contrast, the commonly used β–blocker, propranolol (Prop), inhibited autophagic flux by causing a late block in autophagy. These findings demonstrate a key role for the β_2_-adrenergic receptor in regulating hepatocyte autophagy.

## Results

### The β_2_-agonist, clenbuterol, increases autophagosome number in cultured hepatic cells

**Figure 1:**
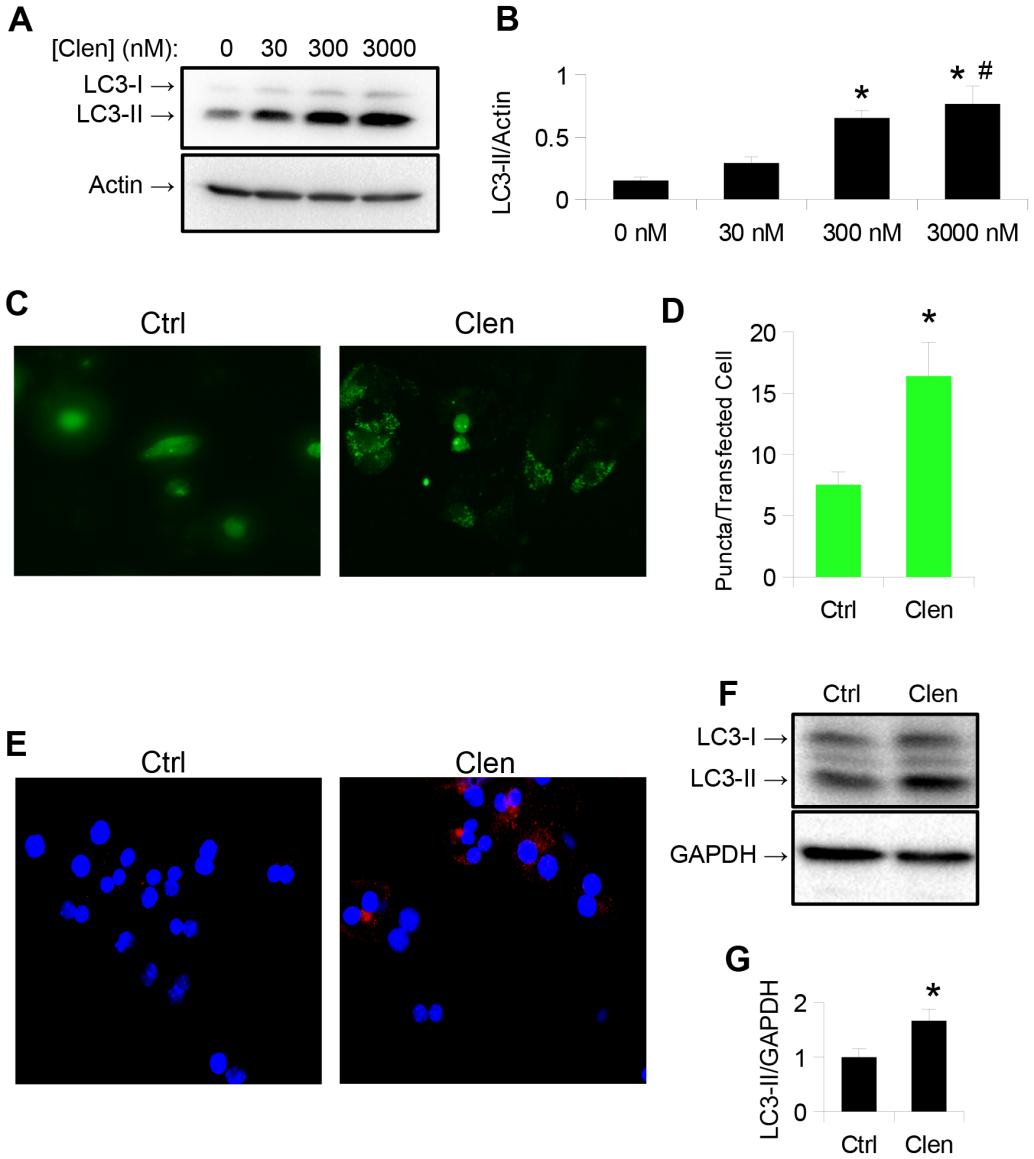
Long acting β_2_-agonist clenbuterol increases autophagosome number in HepG2 cells and in mouse primary hepatocytes. **A–B.**) Clenbuterol increases LC3-II 24 hours after addition in HepG2 cells, at concentrations as low as 300 nM. Asterisk represents significance vs. ctrl, and hash represents significance vs. 30 nM as per Tukey's post-hoc test following one-way ANOVA. **C–D.**) Clenbuterol increases puncta formation in HepG2 cells transiently transfected with eGFP-LC3 plasmid. Image taken at 20× magnification. **E.**) Clenbuterol increases endogenous LC3 puncta in mouse primary hepatocytes. Image taken at 40× magnification, **F–G.**) Clenbuterol increases LC3-II 24 hours after addition in mouse primary hepatocytes. Error bars represent SEM. Unless otherwise noted, asterisk represents p<0.05 relative to control using Student's T-test.

**Figure 2:**
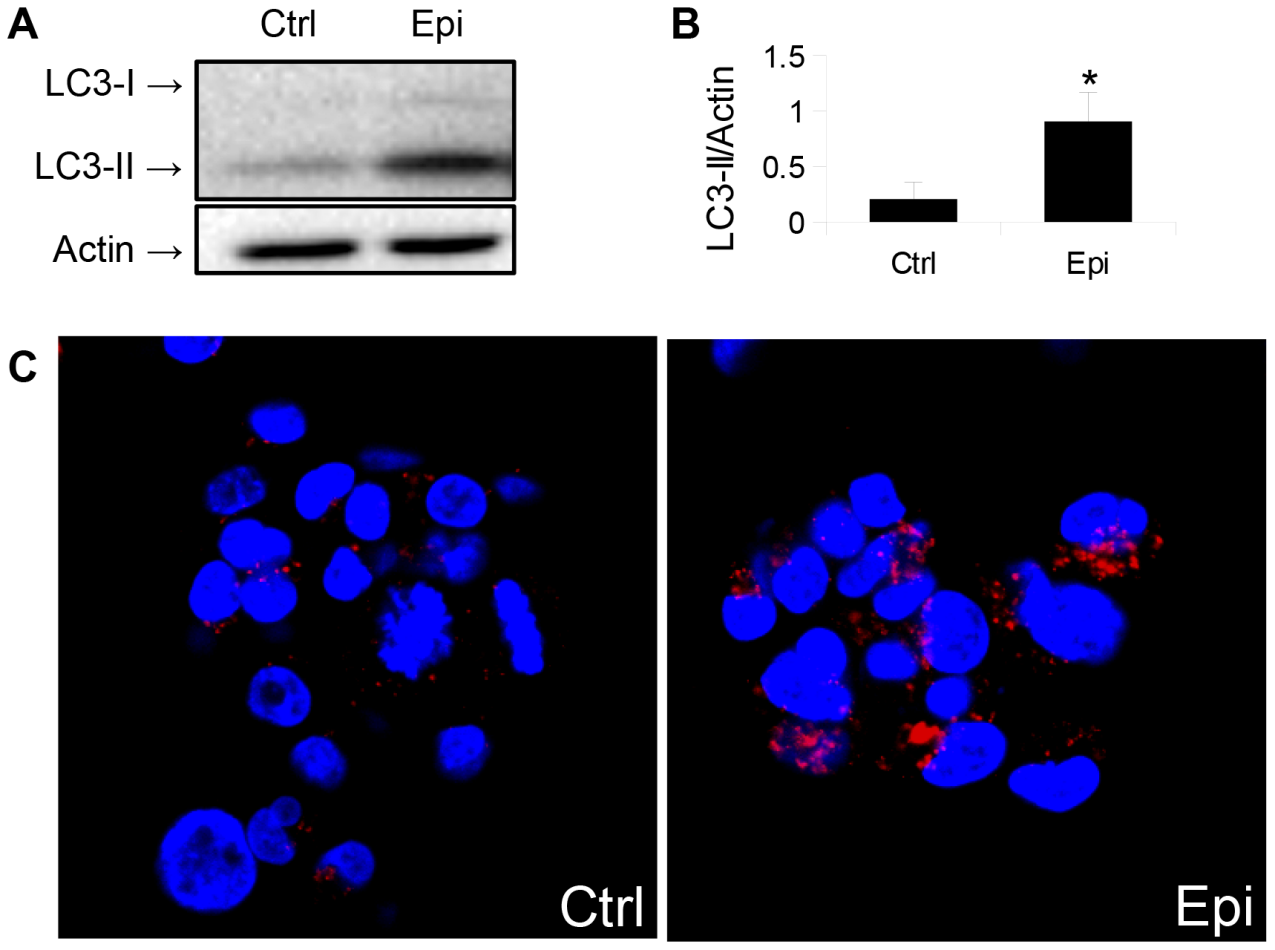
Epinephrine increases autophagosome number in HepG2 cells. **A–B.**) Epinephrine increases LC3-II levels 1 hour after addition in HepG2 cells at 30 nM concentration. **C.**) Epinephrine increases endogenous LC3 puncta in HepG2 cells. For all parts, asterisk indicates p<0.05, error bars represent SEM.

HepG2 cells were chosen to determine the effects of adrenergic stimulation on hepatic autophagy, since they maintain many normal hepatic metabolic functions [20], and clenbuterol was used as a β-agonist since it is clinically approved and has a long half-life. We measured clenbuterol's effect on autophagosome formation by detecting the intracellular level of phosphatidylethanolamine-conjugated microtubule light chain protein 3 (LC3-II), a lipidated autophagosome membrane component that increases with autophagosome formation, and is a commonly used as a marker of autophagosome number [21]. Clenbuterol significantly increased LC3-II levels after 24 hours treatment at concentrations as low as 300 nM (**Fig. 1, A,B**), and this concentration was subsequently used for all further cell culture experiments. Clenbuterol also increased cytosolic LC3 puncta formation when HepG2 cells were transfected with a plasmid expressing LC3 conjugated to green fluorescent protein (GFP) [22]. These findings further suggest increased autophagosome formation. To ensure that these observations were not due to cell line-specific effects, we treated mouse primary hepatocytes with this same dose of clenbuterol for 16 hours, and observed an increase in LC3 immunostaining (**Fig. 1 E**) and LC3-II protein levels, although the amount was less than we found in HepG2 cells (**Fig. 1 F–G**). To check that these effects were not due to a non-specific effect of clenbuterol, we treated HepG2 cells with 30 nM epinephrine for 1 hour. Epinephrine also stimulated an increase in LC3-II protein levels and endogenous LC3-II cytosolic puncta (**Fig. 2**), suggesting that clenbuterol's effects on autophagy were mediated via the β-adrenergic receptor.

### Clenbuterol increases autophagic flux in cultured hepatic cells

**Figure 3:**
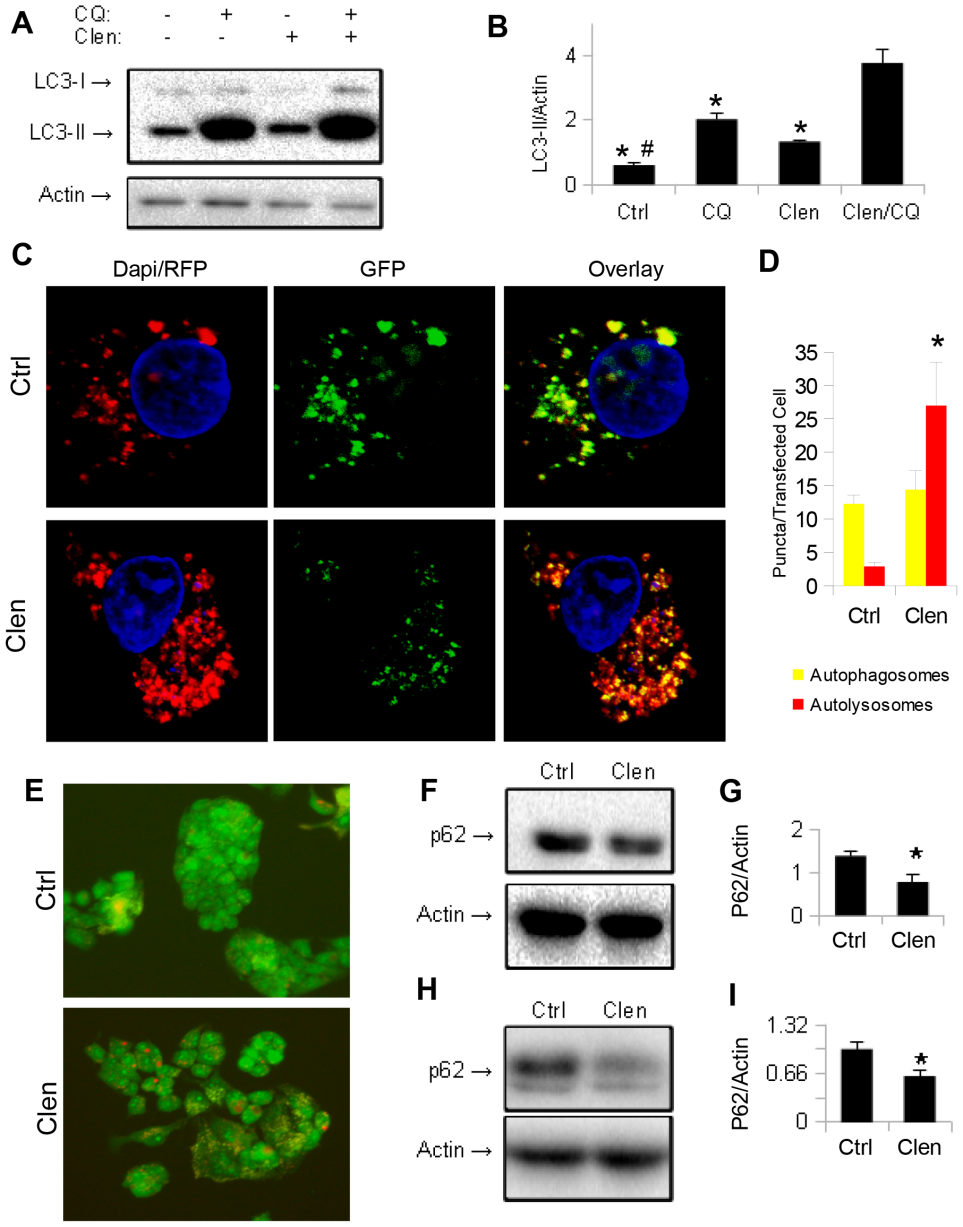
Clenbuterol increases autophagic flux in HepG2 cells and mouse primary hepatocytes. **A–B.**) Co-treatment of HepG2 with Clenbuterol and Chloroquine shows a greater accumulation of LC3-II compared to control cells. Asterisk represents significance vs. Clen/CQ, and hash represents significance vs. CQ as per Tukey's post-hoc test following one-way ANOVA. **C–D.**) Clenbuterol increases autolysosomes in HepG2 cells transiently transfected with GFP-RFP-LC3 plasmid. Image taken at 40× magnification. **E.**) Clenbuterol increases lysosomal acidification (orange/red structures) in HepG2 stained with Acridine Orange. Image taken at 20× magnification. **F–G.**) Clenbuterol induces SQSTM1/p62 degradation in HepG2 cells. **H–I.**) Clenbuterol induces SQSTM1/p62 degradation in mouse primary hepatocytes. Error bars represent SEM. Unless otherwise noted, asterisk represents p<0.05 relative to control using Student's T-test.

Since an increase in LC3-II may be due to either an induction of autophagy or a block at a downstream step [23], cells were co-treated with the lysosomal inhibitor chloroquine. We observed an increase in the LC3-II levels of co-treated cells that was higher than cells treated with chloroquine alone (**Fig. 3 A,B**), consistent with an increase in autophagic flux by clenbuterol [21]. To further confirm an increase in autophagic flux, we transfected cells with a plasmid expressing LC3 fused to GFP and RFP, which emits both green and red fluorescence in autophagosomes, but only red in autolysosomes due to denaturation of the GFP from the acidity within the autolysosome [24]. We saw an increase in both yellow (merged green and red) and red fluorescent puncta following treatment with clenbuterol, indicating an increase in both autophagosomes and autolysosomes (**Fig. 3, C,D**) although only the increase in autolysosomes was statistically significant. Furthermore, clenbuterol increased lysosomal acidity since there was more intense orange fluorescence in clenbuterol-treated HepG2 cells than untreated control HepG2 cells when they were both incubated with the lysotropic dye, acridine orange (**Fig. 3, E**) [25]. Finally, we assessed autophagic flux by measuring the protein level of SQSTM1/P62, a protein which is primarily degraded by autophagy [26]. Clenbuterol treatment induced autophagic flux as it decreased levels of SQSTM1/p62, in both HepG2 cells (**Fig. 3, F, G**) and primary mouse hepatocytes (**Fig. 3, H, I**).

### Clenbuterol induces autophagy in mouse livers

**Figure 4:**
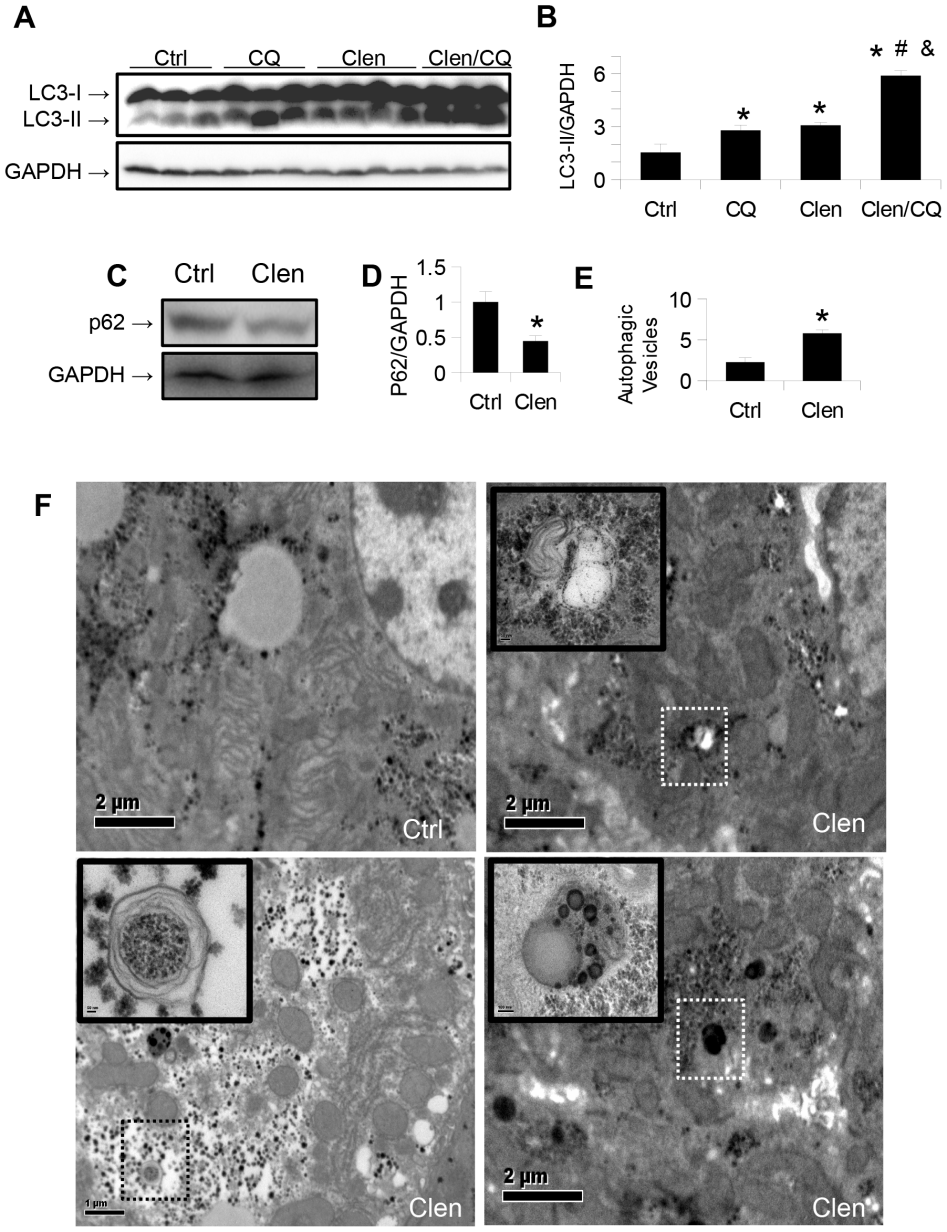
Clenbuterol increases autophagic flux *in vivo*. **A–B.**) Clenbuterol increases autophagosomal marker LC3-II in the liver of mice treated for 3 days. A further increase is seen with co-administration of chloroquine, indicating an increase in autophagic flux. Asterisk represents significance vs. ctrl, hash represents significance vs. CQ, and ampersand represents significance vs. Clen as per Tukey's post-hoc test following one-way ANOVA. **C–D.**) SQSTM1/P62 levels in the livers of the same mice. Asterisk indicates p<0.05. **E–F.**) Electron micrographs of the same mice, showing isolation membranes, autophagosomes, and autolysosomes. A significant increase in the number of autophagic vesicles per cell was observed. Asterisk represents p<0.05. Bar = 2 mm.

To study the effects of β-adrenergic stimulation on hepatic autophagy *in vivo*, we injected clenbuterol *i.p.* into C57BL/6 mice for 3 days. Another group of mice was also injected with the lysosomal inhibitor, chloroquine (CQ) for the same length of time, to block autophagy. Clenbuterol-treated mice had increased hepatic LC3-II levels compared to vehicle-treated control mice (**Fig. 4, A,B**). To rule out non-specific toxic effects, we measured the serum ALT activities in these mice, and found that they were within the normal range (**Table S1 in File S1**). Chloroquine treatment increased LC3-II levels more in the clenbuterol treated mice than in vehicle treated mice, strongly suggesting that the increased LC3-II in the clenbuterol treated mice was due to an increase in autophagic flux, and not from a downstream block in autophagy (**Fig. 4, A,B**) [21]. SQSTM1/p62, was decreased in the clenbuterol treated mice (**Fig. 4, C,D**), further corroborating our findings. Last, demonstration of increased autophagosomes (**Fig. 4 F** **, upper-right and lower-left images**), and autolysosomes (**Fig. 4 F** **, lower-right image**) in the livers of clenbuterol treated mice (**Fig. 4, E, F**) by transmission electron microscopy provided further evidence for induction of autophagic flux by clenbuterol.

### Propranolol treatment inhibits hepatic autophagy even in the absence of agonist

**Figure 5:**
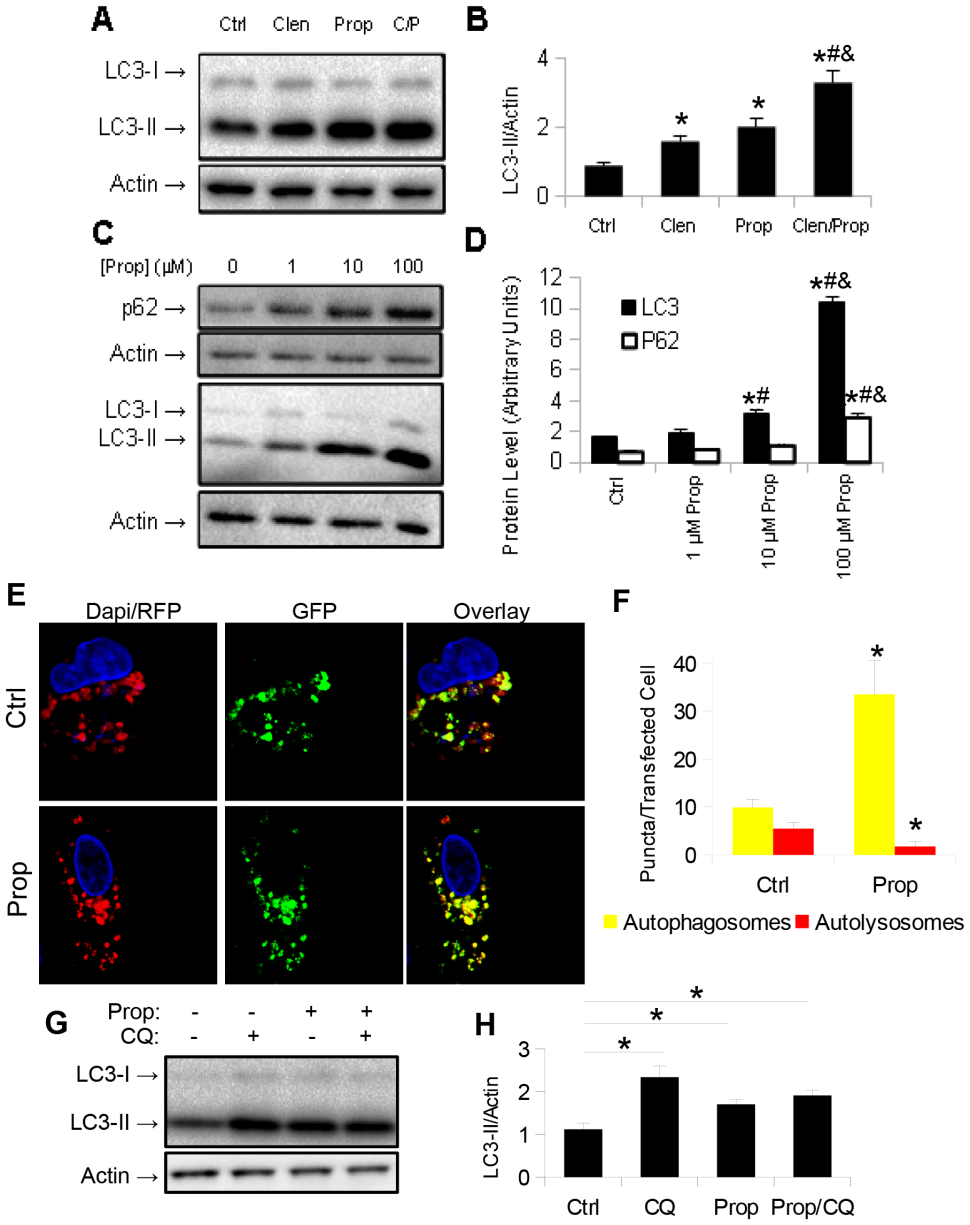
Propranolol inhibits autophagic flux in HepG2 cells. **A–B.**) Propranolol increases LC3-II levels in HepG2 cells, even in the absence of adrenergic agonist. Asterisk represents significance vs. ctrl, hash represents significance vs. Clen, and ampersand represents significance vs. Prop as per Tukey's post-hoc test following one-way ANOVA. **C–D.**) Propranolol inhibits autophagic protein turnover in HepG2 cells. LC3-II and SQSTM1/p62 levels are increased with increasing doses of propranolol. Asterisk represents significance vs. ctrl, hash represents significance vs. 1 µM, and ampersand represents significance vs. 10 µM as per Tukey's post-hoc test following one-way ANOVA. **E–F.**) Propranolol increases autophagosome number, but decreases autolysosome number in HepG2 cells transiently transfected with GFP-RFP-LC3 plasmid. Image taken at 40× magnification. Asterisk represents p<0.05 as per Student's t-test with respect to control. **G–H.**) Co-treatment of HepG2 cells with chloroquine and propranolol shows no increased accumulation of LC3-II compared to control cells treated with chloroquine. Asterisk represents significance vs. ctrl as per Tukey's post-hoc test following one-way ANOVA. For all parts, error bars represent SEM.

**Figure 6:**
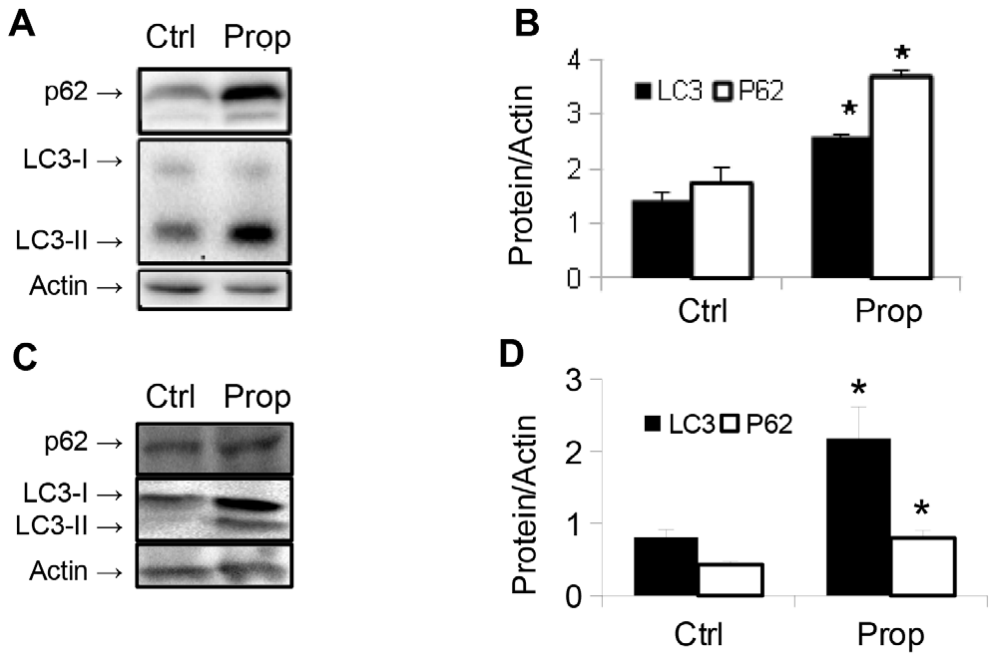
Propranolol inhibits autophagic flux in mouse primary hepatocytes and *in vivo*. Propranolol induces accumulation of both LC3-II and p62 in mouse primary hepatocytes (**A,B**), and in mouse liver (**C,D**). Asterisk represents p<0.05 as per Student's t-test with respect to control. For all parts, error bars represent SEM.

**Figure 7:**
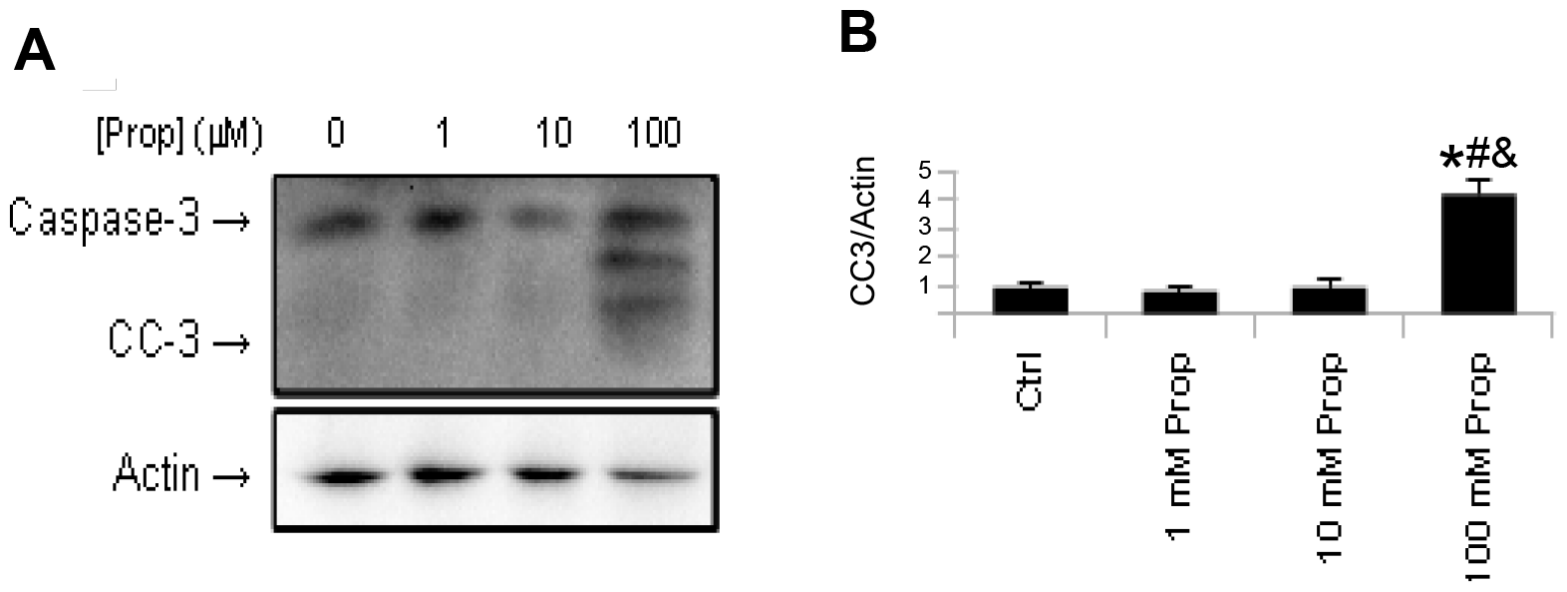
High doses of propranolol increase Caspase-3 cleavage in HepG2 cells. Asterisk represents significance vs. ctrl, hash represents significance vs. 1 µM, and ampersand represents significance vs. 10 µM as per Tukey's post-hoc test following one-way ANOVA. Error bars represent SEM.

To further understand the effects of β-adrenergic actions on hepatic autophagy, we treated HepG2 cells with clenbuterol and the β-blocker, propranolol. As expected, clenbuterol increased LC3-II levels. However, to our surprise, propranolol alone increased LC3-II levels, and treatment with both propranolol and clenbuterol further increased its levels (**Fig. 5, A, B**). When HepG2 cells were treated with increasing doses of propranolol, we observed increases in both LC3-II and SQSTM1/p62 levels by Western blotting (**Fig. 5, C, D**) suggesting that autophagy was blocked at a late stage (such as lysosomal fusion, acidification, or protease action) [21]. To further confirm that propranolol caused a late block in autophagy, we transfected HepG2 cells with the GFP-RFP-LC3 plasmid [24], and observed an increase in autophagosomes (yellow puncta), that was coupled with a decrease in autolysosomes (red puncta) in propranolol treated cells compared to vehicle-treated control cells (**Fig. 5, E, F**). Furthermore, co-treatment of propranolol treated HepG2 cells with the lysosomal inhibitor chloroquine showed no increase in LC3-II levels compared to control HepG2 cells treated with chloroquine, indicating that autophagosome formation was not increased by propranolol (**Fig. 5, G, H**). To further confirm these results, we treated primary mouse hepatocytes with 10 µM propranolol and observed similar increases in LC3-II and SQSTM1/p62 levels (**Fig. 6, A, B**). Finally, we injected male C57BL/6 mice *i.p.* with propranolol (60 mg/kg/day) for three days, and observed an increase in both LC3-II and SQSTM1/p62 levels, indicating a late block in autophagy had occurred in the livers of mice treated with propranolol (**Fig. 6, C, D**). Of note, a small, but statistically significant increase in serum ALT activity was seen in the propranolol treated mice (**Table S1 in File S1**). We also observed cell death and cleavage of caspase-3 (CC-3) at the highest doses given to HepG2 cells (**Fig. 7**), most likely due to the severe block in autophagy that occurred at these doses.

## Discussion

Classically, the adrenergic system is activated in times of stress, and plays an important role in providing energy for the “fight or flight” response. In metabolic stress or starvation, adrenergic stimulation mobilizes and degrades lipids [27], glycogen [28], and proteins [29] to be used for ketone body formation and gluconeogenesis [3]. These metabolic pathways are also subject to regulation by autophagy [3], [4], [30]. Thus, it is likely that adrenergic regulation of these metabolic of pathways in the normal and activated states may be mediated, at least in part, by autophagy. In this connection, recent studies have shown an increase in hepatic steatosis in mice lacking epinephrine [18], as well as in mice that are defective in autophagy [31]. Additionally, adrenergic-stimulated peripheral lipolysis may require autophagy [32].

In this manuscript, we used multiple techniques to investigate the effects of clenbuterol on autophagosome formation and autophagic flux in human hepatoma cells and mouse primary hepatocytes. In particular, we showed that clenbuterol increased intracellular LC3-II protein levels, LC3 puncta formation, autophagosome/lysosome fusion, acidification of lysosomes, degradation of SQSTM1/P62, and demonstrated autophagic features in electron micrographs (**Fig. 1** **–** **3**). Taken together, these data provide strong evidence that β_2_-adrenergic signaling increases autophagic flux in hepatoma cells, primary hepatocytes in culture, and *in vivo*.

Our cell culture and *in vivo* results are consistent with recent findings by Aranguiz-Urroz and colleagues who showed that β_2_-adrenergic stimulation induced autophagy in cardiac fibroblasts [16]. Another recent study also linked an increase in intracellular cAMP to induction of autophagy in fibroblasts [17]. In contrast, previous studies showed cAMP blocked autophagy in yeast and isolated hepatocytes. Additionally, adrenergic signalling appeared to decrease proteolysis only in specific types of skeletal muscle [33]. Therefore, while the reason(s) for these apparent discrepancies is not known, it is possible thet may be due to differences in cell type, culture/diet conditions, or employment of methods before more reliable modern techniques for studying autophagy were developed [12], [13].

The mechanism for clenbuterol induction of autophagy does not seem to involve mTOR signalling since phosphorylated mTOR levels were not reduced after clenbuterol treatment, and instead were increased in mice treated with clenbuterol (**Fig. S1 in File S1**). In contrast, phosphorylated AMPK levels were higher in mice treated with clenbuterol (**Fig. S2 in File S1**). The AMPK pathway, which is pro-autophagic, through its activating phosphorylation of ULK1 [34], can be induced by changes in energy state, intracellular calcium levels, or EPAC1 activation by cAMP [34], [35]. Supporting the latter possibility, PKA inhibitor H89 failed to inhibit autophagy in HepG2 cells (Farah and Yen, unpublished results) suggesting that increased intracellular cAMP by β_2_-adrenergic stimulation may signal through a non-PKA mediated pathway. Interestingly, H89 itself increased autophagic flux by a non-PKA-mediated pathway in mouse neuroblastoma and embryonic fibroblast cells [36]. Further studies are needed to elucidate the details of the molecular mechanism for β-adrenergic induction of autophagy.

Our results with the β-blocker, propranolol, were quite striking, particularly since it inhibited autophagic flux even in the absence of agonist. McKee *et al.* recently showed that propranolol induced hepatic inflammation, steatosis, and apoptosis in mouse livers: however, the mechanism for these changes was not investigated [37]. Interestingly, similar hepatic changes occurred in genetically-engineered mice that were defective in autophagy [31]. Thus, it is possible that the manifestations of NAFLD caused by toxic effects of the high doses of propranolol observed by McKee *et al.* [37] were due to inhibition of autophagy. Further studies that examine autophagic flux in the setting of NAFLD and propranolol treatment will help establish whether there indeed is such a causal link.

Propranolol previously was shown to act as an inverse agonist in the heart [11], and thus would be expected to reduce autophagosome formation rather than block it at a later stage of autophagy. Our findings showed that propranolol blocked hepatic autophagy in the absence of agonist, so it had its own intrinsic activity in addition to acting as a β-adrenergic antagonist against clenbuterol-induced hepatic autophagy. Since we observed a further increase in LC3II levels after propranolol co-treatment with clenbuterol (**Figs. 5** **,** **6**), it is possible that propranolol accumulates in the lysosome and directly decreases lysosomal activity in a manner similar to chloroquine. Supporting this hypothesis, previous work has shown that lipophilic amines, such as propranolol, can enter the lysosome easily, and raise the pH of that compartment [38]–[40]. However, our data do not preclude the possibilities of propranolol having direct effects on the β-adrenergic receptor, or the existence of a second, as of yet uncharacterised propranolol-interacting protein, that mediates its anti-autophagic effects via an alternative pathway.

Recent research has shown the importance of autophagy in NAFLD [41]–[43], chronic hepatic fibrotic disorders [44], ethanol mediated hepatosteatosis and toxicity [45], and in the prevention of hepatocellular carcinoma [6], [31], [46]. Additionally, mice that overexpress ATG5 and thus have higher levels of macroautophagy, exhibit resistance to the age-related increase in lipids and a decreased GSH/GSSG ratio in their livers [47]. Furthermore, restoration of the age-related loss of chaperone-mediated autophagy improves liver function in aged mice [48]. In contrast, previous studies have linked hepatic autophagy with increased stellate cell activation and liver fibrosis in mice treated with CCl_4_ [7]–[9]. Thus, in disorders that are driven primarily by stellate cell activation, β-blockers may help reduce fibrosis, whereas in other cases in which hepatocyte autophagy is crucial, such as α1 antitrypsin deficiency and hepatosteatosis, β-blockers may cause harmful effects through their inhibition of autophagy.

It is interesting that although β-blockers have been used widely for decades, the impact of β-blockers on NAFLD in the clinical setting has not been reported in the literature. Recently, Serste *et al.* showed that β-blockers were associated with increased mortality in patients with hepatic failure [49]. Additionally, in a large cohort twin study, β-2 adrenergic receptor genotype correlated with plasma levels of gamma-glutamyl transferase and triglycerides, suggesting that it may modulate susceptibility to NAFLD and metabolic syndrome [50].

In summary, we have used multiple methods to show that the β_2_-adrenergic agonist, clenbuterol, induced autophagic flux in cultured human hepatic cells, primary mouse hepatocytes, and mouse liver. In contrast, the β-blocker, propranolol, caused a late block in autophagy. Taken together, these findings help clarify the role of adrenergic receptor signalling in hepatic autophagy.

## Materials and Methods

### Ethics Statement

Study was carried out in strict accordance to the standards described in NIH published *Guide for the*

### Care and Use of Laboratory Animals

Studies were approved by the IACUC at Duke-NUS Graduate Medical School. All reasonable steps to prevent animal suffering were undertaken.

### Reagents

Clenbuterol (C5423), acridine orange (A9231), chloroquine (C6628), glutaraldehyde (G7651), paraformaldehyde (158127), propranolol (P0884), fetal bovine serum (12003C) and dapi (32670) were purchased from Sigma-Aldrich. Cell culture media (11965) was purchased from Invitrogen. Antibodies recognizing LC3 (2775), GAPDH (2118), Cleaved Caspase 3 (9664), and SQSTM1/P62 (5114) were purchased from Cell Signalling Technologies, whereas antibodies recognizing β-actin (sc-8432) as well as HRP conjugated secondary antibodies recognizing mouse (sc-2954) and rabbit (sc-2955) IgG were purchased from SantaCruz Biotechnologies. GFP-RFP-LC3 and GFP-LC3 (Addgene plasmid 21073) plasmids were a gift from T. Yoshimori (Osaka Uni., Osaka, Japan) [22], [24].

### Cell Culture

HepG2 (HB-8065) cells were purchased from ATCC and maintained at 37°C in DMEM containing 10% FBS in a 5% CO_2_ atmosphere. For GFP-RFP-LC3 and GFP-LC3 experiments, cells were transfected 24 hours prior to the experiment using Lipofectamine 2000 (Invitrogen).

### Primary hepatocyte isolation

Primary mouse hepatocytes were isolated and cultured using a modification of the protocol published by Klaunig et. al [51]. Hepatocytes were isolated from the liver of C57BL/6 mice which were fasted overnight, followed by two-stage collagenase perfusion with HEPES buffer. Cell viability was assessed by trypan blue dye exclusion one hour after isolation. Only preparations with cell viability greater than 95% were used for subsequent experiments. Hepatocytes were treated within 8 hours of isolation, and assayed with 24 hours of isolation. Hepatocytes were maintained in high glucose DMEM also containing 10% FBS and 1× Penicillin/Streptomycin at 37°C in a 5% CO_2_ atmosphere. At the time of assay (24 hours following plating), RNA was extracted from untreated hepatocytes grown on a separate plate, and hepatocyte markers were assessed by quantitative-real time PCR (**Table S2 in File S1**).

### Animal models

Male C57BL/6 mice (8 weeks old) were obtained from NUSCARE and InVivos. Mice had access to food and water *ad libum*. Clenbuterol (1 mg/kg body weight), propranolol (60 mg/kg body weight) and chloroquine (40 mg/kg body weight) were injected in dH_2_O daily *i.p.*, while control mice were injected with dH_2_O vehicle. Animals were sacrificed by CO_2_ inhalation and tissues snap frozen in liquid nitrogen, except those used for electron microscopy, which were preserved in sodium phosphate buffer containing 3% glutyaraldehyde and 2% paraformaldehyde.

### Western blotting

Cultured cells were lysed in mammalian lysis buffer (Sigma-Aldrich) while tissue samples were homogenized by MagNA Lyser (Roche) in the same buffer. Concentration of protein was determined by the BCA Kit (Bio-Rad). Protein was stored at −80°C. Immediately prior to western blotting, Laemmli sample buffer was added (250 mmol/l Tris, pH 7.4, 2% w/v SDS, 25% v/v glycerol, 10% v/v 2-mercaptoethanol, and 0.01% w/v bromophenol blue), and samples were heated to 105°C for 5 minutes, chilled at 4°C for 10 minutes, and immediately ran on an SDS-polyacrylamide gel. Proteins were transferred to a polyvinylidine difluoride membrane (Bio-Rad) in transfer buffer containing 25 mmol/l Tris, pH 8.8, 192 mmol/l glycine, and 10% v/v methanol. All washing, blocking and antibody solutions were prepared in PBST. Membranes were blocked in 5% milk, followed by overnight incubation with primary antibodies in 1% bovine serum albumin. Membranes were washed three times, followed by secondary antibody incubation for 1 hr in 1% bovine serum albumin. Blots were again washed 3 times, and then probed using an enhanced chemiluminescence system (GE Healthcare) on a GelDoc imager (Bio-Rad). Densitometry was performed following acquisition using ImageJ software (NIH).

### Acridine orange staining

Cells were grown in 6-well culture dishes for 24 hours before clenbuterol was added for 24 hours. Cells were then incubated for 15 minutes in PBS containing 1 µg/ml acridine orange, and immediately visualized.

### Immunofluoresence

Prior to treatment, cells were seeded on glass coverslips. Following treatment, cells were washed in PBS, fixed for 15 minutes in 4% formaldehyde, and washed again. Cells were then permeabilized in 100% methanol at −20°C for 10 minutes, washed, and blocked in PBST containing 1% normal goat serum for 1 hour. Cells were then incubated with primary antibody overnight at 4°C, washed thrice with PBS, and then incubated for 2 hours at room temperature with Alexa Fluor secondary antibodies (Invitrogen). Cells were washed once, and then treated with dapi at 1∶3000 dilution in PBS for 15 minutes. Coverslips were mounted using Vectashield mounting media (Invitrogen), and visualized using an LSM710 Carl Zeiss confocal microscope.

### Electron Microscopy

Fresh tissue was placed in fixative containing 2% paraformaldehyde and 3% gluteraldehyde in pH 7.4 phosphate buffer overnight at 4°C. Tissue was washed once in PBS, followed by post-fixation with 1% osmium tetroxide. Samples were dehydrated in washes with ascending concentrations of alcohol, followed by embedding in Araldite. Ultra-thin sections were cut and stained with uranyl acetate and lead citrate. Imaging was performed on an Olympus EM208S transmission electron microscope. Autophagic vesicles were defined as autophagosomes (double-membraned structures surrounding cytoplasmic material) and autolysosomes (lysosomes containing cytoplasmic material). Vesicles were counted in 10 random fields per mouse, in 3 mice per condition.

### ALT Activity Assay

ALT Activity was measured in mouse serum collected by cardiac puncture at time of death using Cayman Chemical's ALT activity kit (700260). All manufacturer's instructions were followed.

### mRNA isolation and quantitative real-time PCR

RNA was isolated from cells by the Invitek Mini Kit (Invitek) or from tissues by TriZOL (Sigma-Aldrich). 1 µg RNA was reverse-transcribed according to the manufacturer's instructions using the iScript Select cDNA Synthesis Kit (Bio-Rad). Quantitative Real-Time PCR was performed using the QuantiTect SYBR Green Kit (QIAGEN). Cycolophilin A expression was used for normalization, while fold change was calculated using 2^−ΔΔCt^. Primer sequences are available upon request.

### Statistics

Cell culture experiments were performed in triplicates and repeated 3 independent times using matched controls. Results were expressed as mean ± SEM. For figures 1G, 2B, 3G, 3I, 4D, 4E, 5F, 6B, and 6D, as well as all supplemental figures and tables, statistical significance was calculated using Student's t-test, taking p<0.05 as significant. For figures 1B, 3B, 4B, 5B, 5D, 5H, and 7B, one-way ANOVA was performed, followed by Tukey's post-hoc test between all groups, with p<0.05 taken as significant.

## Supporting Information

Figure S1 **Clenbuterol increases mTOR phosphorylation in mouse liver.** n = 5, asterisk represents p<0.05.
(TIF)

Figure S2 **Clenbuterol increases AMPK phosphorylation in mouse liver.** n = 5, asterisk represents p<0.05.
(TIF)

File S1 **Table S1**, Serum ALT activity in mice treated for 3 days with 1 mg/kg clenbuterol or 60 mg/kg propranolol. **Table S2**, Expression of hepatocyte marker genes in isolated hepatocytes, normal mouse liver, and normal mouse heart.
(DOCX)

## References

[1] Meschini S, Condello M, Lista P, Arancia G (2011) Autophagy: Molecular mechanisms and their implications for anticancer therapies. Curr Cancer Drug Targets 11: 357–379. 21247381 10.2174/156800911794519707

[2] Mizushima N, Komatsu M (2011) Autophagy: renovation of cells and tissues. Cell 147: 728–741. 22078875 10.1016/j.cell.2011.10.026

[3] Ezaki J, Matsumoto N, Takeda-Ezaki M, Komatsu M, Takahashi K, et al (2011) Liver autophagy contributes to the maintenance of blood glucose and amino acid levels. Autophagy 7: 727–736. 21471734 10.4161/auto.7.7.15371 PMC3149698

[4] Singh R, Kaushik S, Wang Y, Xiang Y, Novak I, et al (2009) Autophagy regulates lipid metabolism. Nature 458: 1131–1135. 19339967 10.1038/nature07976 PMC2676208

[5] Sinha RA, You SH, Zhou J, Siddique MM, Bay BH, et al (2012) Thyroid hormone stimulates hepatic lipid catabolism via activation of autophagy. J Clin Invest 122: 2428–2438. 22684107 10.1172/JCI60580 PMC3386813

[6] Komatsu M, Kurokawa H, Waguri S, Taguchi K, Kobayashi A, et al (2010) The selective autophagy substrate p62 activates the stress responsive transcription factor Nrf2 through inactivation of Keap1. Nat Cell Biol 12: 213–223. 20173742 10.1038/ncb2021

[7] Thoen LF, Guimaraes EL, Dolle L, Mannaerts I, Najimi M, et al (2011) A role for autophagy during hepatic stellate cell activation. J Hepatol 55: 1353–1360. 21803012 10.1016/j.jhep.2011.07.010

[8] Hernandez-Gea V, Ghiassi-Nejad Z, Rozenfeld R, Gordon R, Fiel MI, et al (2012) Autophagy releases lipid that promotes fibrogenesis by activated hepatic stellate cells in mice and in human tissues. Gastroenterology 142: 938–946. 22240484 10.1053/j.gastro.2011.12.044 PMC3439519

[9] Hernandez-Gea V, Hilscher M, Rozenfeld R, Lim MP, Nieto N, et al (2013) Endoplasmic reticulum stress induces fibrogenic activity in hepatic stellate cells through autophagy. J Hepatol 59: 98–104. 23485523 10.1016/j.jhep.2013.02.016 PMC3686909

[10] Boner AL, Vallone G, Brighenti C, Schiassi M, Miglioranzi P, et al (1988) Comparison of the protective effect and duration of action of orally administered clenbuterol and salbutamol on exercise-induced asthma in children. Pediatr Pulmonol 4: 197–200. 3393382 10.1002/ppul.1950040402

[11] Wachter SB, Gilbert EM (2012) Beta-adrenergic receptors, from their discovery and characterization through their manipulation to beneficial clinical application. Cardiology 122: 104–112. 22759389 10.1159/000339271

[12] Holen I, Gordon PB, Seglen PO (1991) Role of cyclic nucleotides in the control of hepatic autophagy. Biomed Biochim Acta 50: 389–392. 1666282

[13] Schmelzle T, Beck T, Martin DE, Hall MN (2004) Activation of the RAS/cyclic AMP pathway suppresses a TOR deficiency in yeast. Mol Cell Biol 24: 338–351. 14673167 10.1128/MCB.24.1.338-351.2004 PMC303340

[14] Gordon PB, Holen I, Seglen PO (1991) Effects of adrenergic agonists and antagonists on autophagic activity in isolated rat liver cells. Biomed Biochim Acta 50: 383–387. 1801702

[15] Kondomerkos DJ, Kalamidas SA, Kotoulas OB, Hann AC (2005) Glycogen autophagy in the liver and heart of newborn rats. The effects of glucagon, adrenalin or rapamycin. Histol Histopathol 20: 689–696. 15944916 10.14670/HH-20.689

[16] Aranguiz-Urroz P, Canales J, Copaja M, Troncoso R, Vicencio JM, et al (2011) Beta(2)-adrenergic receptor regulates cardiac fibroblast autophagy and collagen degradation. Biochim Biophys Acta 1812: 23–31. 20637865 10.1016/j.bbadis.2010.07.003

[17] Ugland H, Naderi S, Brech A, Collas P, Blomhoff HK (2011) cAMP induces autophagy via a novel pathway involving ERK, cyclin E and Beclin 1. Autophagy 7: 1199–1211. 21750416 10.4161/auto.7.10.16649

[18] Sharara-Chami RI, Zhou Y, Ebert S, Pacak K, Ozcan U, et al (2012) Epinephrine deficiency results in intact glucose counter-regulation, severe hepatic steatosis and possible defective autophagy in fasting mice. Int J Biochem Cell Biol 44: 905–913. 22405854 10.1016/j.biocel.2012.02.016 PMC4710484

[19] Baker JG (2010) The selectivity of beta-adrenoceptor agonists at human beta1-, beta2- and beta3-adrenoceptors. Br J Pharmacol 160: 1048–1061. 20590599 10.1111/j.1476-5381.2010.00754.x PMC2936015

[20] Javitt NB (1990) Hep G2 cells as a resource for metabolic studies: lipoprotein, cholesterol, and bile acids. FASEB J 4: 161–168. 2153592 10.1096/fasebj.4.2.2153592

[21] Klionsky DJ, Abdalla FC, Abeliovich H, Abraham RT, Acevedo-Arozena A, et al (2012) Guidelines for the use and interpretation of assays for monitoring autophagy. Autophagy 8: 445–544. 22966490 10.4161/auto.19496 PMC3404883

[22] Kabeya Y, Mizushima N, Ueno T, Yamamoto A, Kirisako T, et al (2000) LC3, a mammalian homologue of yeast Apg8p, is localized in autophagosome membranes after processing. EMBO J 19: 5720–5728. 11060023 10.1093/emboj/19.21.5720 PMC305793

[23] Mizushima N, Yoshimori T, Levine B (2010) Methods in mammalian autophagy research. Cell 140: 313–326. 20144757 10.1016/j.cell.2010.01.028 PMC2852113

[24] Kamimoto T, Shoji S, Hidvegi T, Mizushima N, Umebayashi K, et al (2006) Intracellular inclusions containing mutant alpha1-antitrypsin Z are propagated in the absence of autophagic activity. J Biol Chem 281: 4467–4476. 16365039 10.1074/jbc.M509409200

[25] Traganos F, Darzynkiewicz Z (1994) Lysosomal proton pump activity: supravital cell staining with acridine orange differentiates leukocyte subpopulations. Methods Cell Biol 41: 185–194. 7532261 10.1016/s0091-679x(08)61717-3

[26] Bjorkoy G, Lamark T, Pankiv S, Overvatn A, Brech A, et al (2009) Monitoring autophagic degradation of p62/SQSTM1. Methods Enzymol 452: 181–197. 19200883 10.1016/S0076-6879(08)03612-4

[27] Lampidonis AD, Rogdakis E, Voutsinas GE, Stravopodis DJ (2011) The resurgence of Hormone-Sensitive Lipase (HSL) in mammalian lipolysis. Gene 477: 1–11. 21241784 10.1016/j.gene.2011.01.007

[28] Brodde OE (1990) Physiology and pharmacology of cardiovascular catecholamine receptors: implications for treatment of chronic heart failure. Am Heart J 120: 1565–1572. 2248213 10.1016/0002-8703(90)90060-b

[29] Pereira C, Murphy K, Jeschke M, Herndon DN (2005) Post burn muscle wasting and the effects of treatments. Int J Biochem Cell Biol 37: 1948–1961. 16109499 10.1016/j.biocel.2005.05.009

[30] Kotoulas OB, Kalamidas SA, Kondomerkos DJ (2006) Glycogen autophagy in glucose homeostasis. Pathol Res Pract 202: 631–638. 16781826 10.1016/j.prp.2006.04.001

[31] Takamura A, Komatsu M, Hara T, Sakamoto A, Kishi C, et al (2011) Autophagy-deficient mice develop multiple liver tumors. Genes Dev 25: 795–800. 21498569 10.1101/gad.2016211 PMC3078705

[32] Lizaso A, Tan KT, Lee YH (2013) beta-adrenergic receptor-stimulated lipolysis requires the RAB7-mediated autolysosomal lipid degradation. Autophagy 9: 1228–1243. 23708524 10.4161/auto.24893 PMC3748194

[33] Goncalves DA, Silveira WA, Lira EC, Graca FA, Paula-Gomes S, et al (2012) Clenbuterol suppresses proteasomal and lysosomal proteolysis and atrophy-related genes in denervated rat soleus muscles independently of Akt. Am J Physiol Endocrinol Metab 302: E123–133. 21952035 10.1152/ajpendo.00188.2011

[34] Alers S, Loffler AS, Wesselborg S, Stork B (2012) Role of AMPK-mTOR-Ulk1/2 in the regulation of autophagy: cross talk, shortcuts, and feedbacks. Mol Cell Biol 32: 2–11. 22025673 10.1128/MCB.06159-11 PMC3255710

[35] Omar B, Zmuda-Trzebiatowska E, Manganiello V, Goransson O, Degerman E (2009) Regulation of AMP-activated protein kinase by cAMP in adipocytes: roles for phosphodiesterases, protein kinase B, protein kinase A, Epac and lipolysis. Cell Signal 21: 760–766. 19167487 10.1016/j.cellsig.2009.01.015 PMC3576575

[36] Inoue H, Hase K, Segawa A, Takita T (2013) H89 (N-[2-p-bromocinnamylamino-ethyl]-5-isoquinolinesulphonamide) induces autophagy independently of protein kinase A inhibition. Eur J Pharmacol 714: 170–177. 23810683 10.1016/j.ejphar.2013.06.018

[37] McKee C, Soeda J, Asilmaz E, Sigalla B, Morgan M, et al (2013) Propranolol, a beta-adrenoceptor antagonist, worsens liver injury in a model of non-alcoholic steatohepatitis. Biochem Biophys Res Commun 437: 597–602. 23850676 10.1016/j.bbrc.2013.07.005 PMC5226920

[38] Ishizaki J, Yokogawa K, Ichimura F, Ohkuma S (2000) Uptake of imipramine in rat liver lysosomes in vitro and its inhibition by basic drugs. J Pharmacol Exp Ther 294: 1088–1098. 10945864

[39] Kazmi F, Hensley T, Pope C, Funk RS, Loewen GJ, et al (2013) Lysosomal sequestration (trapping) of lipophilic amine (cationic amphiphilic) drugs in immortalized human hepatocytes (Fa2N-4 cells). Drug Metab Dispos 41: 897–905. 23378628 10.1124/dmd.112.050054 PMC3608459

[40] Ashoor R, Yafawi R, Jessen B, Lu S (2013) The contribution of lysosomotropism to autophagy perturbation. PLoS One 8: e82481. 24278483 10.1371/journal.pone.0082481 PMC3838419

[41] Stankov MV, Panayotova-Dimitrova D, Leverkus M, Vondran FW, Bauerfeind R, et al (2012) Autophagy inhibition due to thymidine analogues as novel mechanism leading to hepatocyte dysfunction and lipid accumulation. AIDS 26: 1995–2006. 22914580 10.1097/QAD.0b013e32835804f9

[42] Lin CW, Zhang H, Li M, Xiong X, Chen X, et al (2013) Pharmacological promotion of autophagy alleviates steatosis and injury in alcoholic and non-alcoholic fatty liver conditions in mice. J Hepatol 58: 993–999. 23339953 10.1016/j.jhep.2013.01.011 PMC3634371

[43] Sinha RA, Farah BL, Singh BK, Siddique MM, Li Y, et al (2013) Caffeine stimulates hepatic lipid metabolism via autophagy-lysosomal pathway. Hepatology 10.1002/hep.26667 23929677

[44] Hidvegi T, Ewing M, Hale P, Dippold C, Beckett C, et al (2010) An autophagy-enhancing drug promotes degradation of mutant alpha1-antitrypsin Z and reduces hepatic fibrosis. Science 329: 229–232. 20522742 10.1126/science.1190354

[45] Ding WX, Li M, Chen X, Ni HM, Lin CW, et al (2010) Autophagy reduces acute ethanol-induced hepatotoxicity and steatosis in mice. Gastroenterology 139: 1740–1752. 20659474 10.1053/j.gastro.2010.07.041 PMC4129642

[46] Kotsafti A, Farinati F, Cardin R, Cillo U, Nitti D, et al (2012) Autophagy and apoptosis-related genes in chronic liver disease and hepatocellular carcinoma. BMC Gastroenterol 12: 118. 22928777 10.1186/1471-230X-12-118 PMC3449193

[47] Pyo JO, Yoo SM, Ahn HH, Nah J, Hong SH, et al (2013) Overexpression of Atg5 in mice activates autophagy and extends lifespan. Nat Commun 4: 2300. 23939249 10.1038/ncomms3300 PMC3753544

[48] Zhang C, Cuervo AM (2008) Restoration of chaperone-mediated autophagy in aging liver improves cellular maintenance and hepatic function. Nat Med 14: 959–965. 18690243 10.1038/nm.1851 PMC2722716

[49] Serste T, Melot C, Francoz C, Durand F, Rautou PE, et al (2010) Deleterious effects of beta-blockers on survival in patients with cirrhosis and refractory ascites. Hepatology 52: 1017–1022. 20583214 10.1002/hep.23775

[50] Loomba R, Rao F, Zhang L, Khandrika S, Ziegler MG, et al (2010) Genetic covariance between gamma-glutamyl transpeptidase and fatty liver risk factors: role of beta2-adrenergic receptor genetic variation in twins. Gastroenterology 139: 836–845. 20537997 10.1053/j.gastro.2010.06.009 PMC3038676

[51] Klaunig JE, Goldblatt PJ, Hinton DE, Lipsky MM, Chacko J, et al (1981) Mouse liver cell culture. I. Hepatocyte isolation. In Vitro 17: 913–925. 6273298 10.1007/BF02618288

