# Supplementary figures and images for: β-Adrenergic Agonist and Antagonist Regulation of Autophagy in HepG2 Cells, Primary Mouse Hepatocytes, and Mouse Liver
Source: PLoS One. 2014 Jun 20;9(6):e98155. doi: 10.1371/journal.pone.0098155 (PMC4064960; doi:10.1371/journal.pone.0098155)

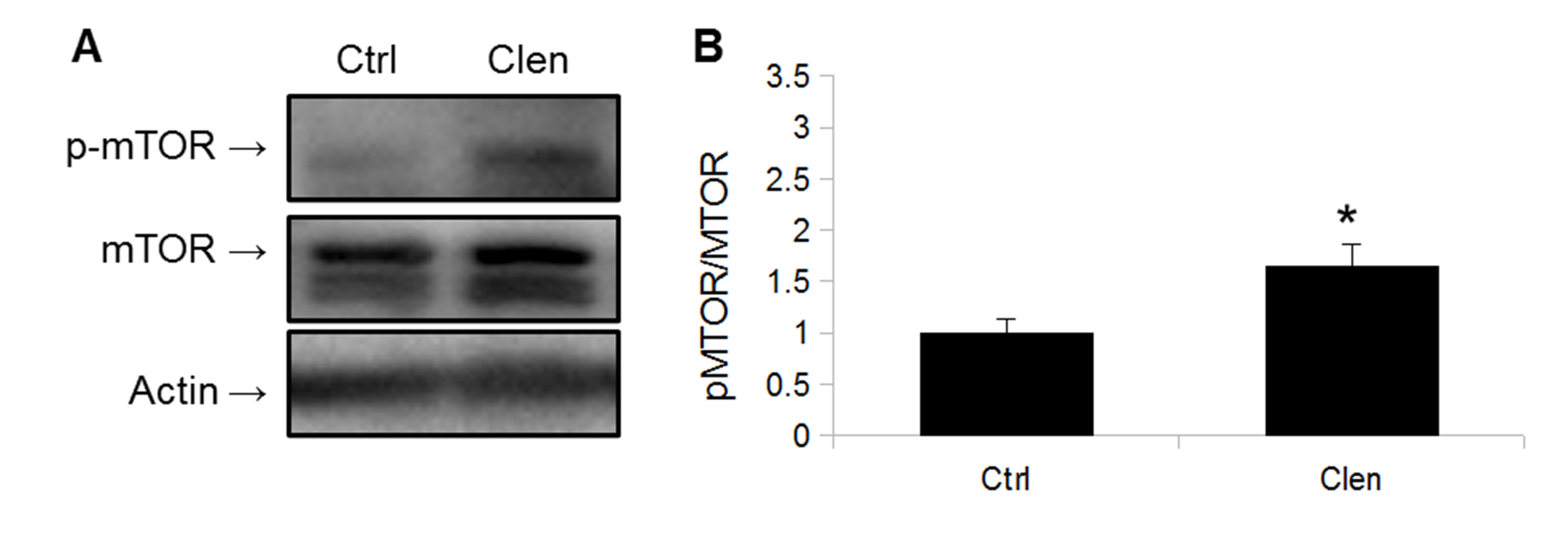

Supplement: Figure S1 — Clenbuterol increases mTOR phosphorylation in mouse liver. n = 5, asterisk represents p<0.05. (TIF) [file pone.0098155.s001.tif]

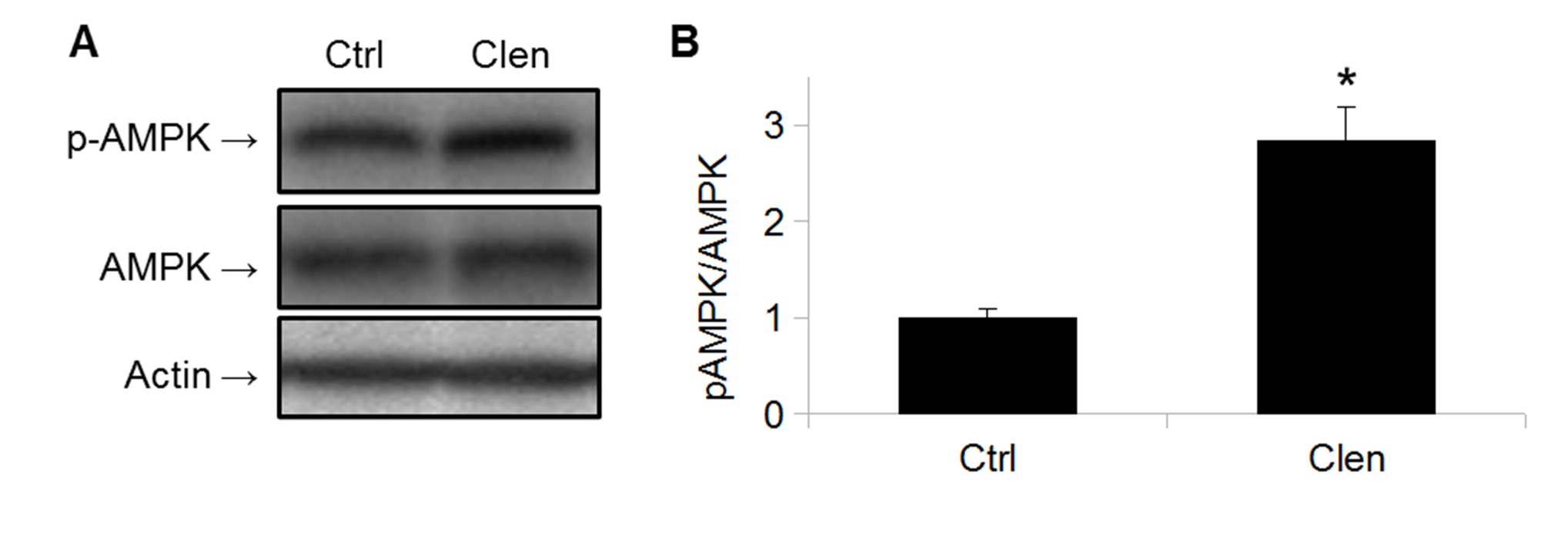

Supplement: Figure S2 — Clenbuterol increases AMPK phosphorylation in mouse liver. n = 5, asterisk represents p<0.05. (TIF) [file pone.0098155.s002.tif]
