# Supplementary material for: β-Adrenergic Agonist and Antagonist Regulation of Autophagy in HepG2 Cells, Primary Mouse Hepatocytes, and Mouse Liver
Source: PLoS One. 2014 Jun 20;9(6):e98155. doi: 10.1371/journal.pone.0098155 (PMC4064960; doi:10.1371/journal.pone.0098155)
Supplement: File S1 — Table S1, Serum ALT activity in mice treated for 3 days with 1 mg/kg clenbuterol or 60 mg/kg propranolol. Table S2, Expression of hepatocyte marker genes in isolated hepatocytes, normal mouse liver, and normal mouse heart. (DOCX) [file pone.0098155.s003.docx]

**Supporting Figure Legends**

**Figure S1**: Clenbuterol increases mTOR phosphorylation in mouse liver. n=5, asterisk represents p<0.05.

**Figure S2**: Clenbuterol increases AMPK phosphorylation in mouse liver. n=5, asterisk represents p<0.05.

**Supporting tables:**

**Table S1: Serum ALT activity in mice treated for 3 days with 1 mg/kg clenbuterol or 60 mg/kg propranolol.**

|  | **Control** | **Clenbuterol** | **Propranolol** |
| --- | --- | --- | --- |
| *ALT Activity* | 14.62+3.285 | 21.31+5.553 | 48.30+25.56 |
| *p-value* |  | 0.0632 | 0.0415 |

Values are expressed as activity (U/L) + SD. Values less than 40 U/L are accepted to be within the normal range for mouse serum, those above are indicated in red. n = 5 for all groups. p-value is calculated using Student's t-test, relative to control.

**Supplemental Table 2: Expression of hepatocyte marker genes in isolated hepatocytes, normal mouse liver, and normal mouse heart.**

| **Gene** | **Hepatocyte** | **Liver** | **Heart** | **p-value (Hepatocyte-Heart)** |
| --- | --- | --- | --- | --- |
| *Albumin* | 0.4493+0.0475 | 1.0000+0.0728 | 0.0007+0.00002 | 0.011 |
| *Cyp3A11* | 0.0397+0.0038 | 1.0000+0.1742 | 0.0003+0.0001 | 0.009 |
| *HNF4a* | 0.0542+0.0060 | 1.0000+0.2093 | 0.0020+0.0006 | 0.012 |

Values are expressed as fold change relative to normal liver + SEM. n=3 for hepatocytes, n=4 for liver and heart. p-value is calculated using Student's t-test between primary hepatocytes and heart.
